# Incidental intraoperative finding of jejunal neuroendocrine tumors during elective ventral hernia repair

**DOI:** 10.1093/jscr/rjad530

**Published:** 2023-09-24

**Authors:** Gasinee Reed, David Kim, Kelsey Hayes, Richard Wirz

**Affiliations:** Department of General Surgery, Poplar Bluff Regional Medical Center, Poplar Bluff, MO 63901, United States; Department of General Surgery, Poplar Bluff Regional Medical Center, Poplar Bluff, MO 63901, United States; Department of General Surgery, Poplar Bluff Regional Medical Center, Poplar Bluff, MO 63901, United States; Department of General Surgery, Poplar Bluff Regional Medical Center, Poplar Bluff, MO 63901, United States

**Keywords:** surgery, gastroenterology, oncology, ventral hernia, neuroendocrine tumors

## Abstract

Neuroendocrine tumors (NETs) constitute ~0.5% of all diagnosed malignancies. In our case, a 72-year-old male, who was asymptomatic aside from mild left lower abdominal pain, was scheduled for elective ventral hernia repair, evident on computed tomography. The laparoscopic ventral hernia repair necessitated the conversion to laparotomy due to extensive adhesions and the incorporation of surgical mesh into the small bowel wall. The patient suffered from delayed small bowel injury resulting in the second emergent laparotomy when numerous calcified lesions were incidentally noted in the small bowel wall. Pathology confirmed Grade 1 well-differentiated NETs of the jejunum. This case highlights the importance of considering NETs as part of a differential diagnosis in patients with nonspecific symptoms and negative imaging studies. This case also emphasizes the importance of early detection of this rare pathology to improve prognosis and outcome.

## Introduction

Neuroendocrine tumors (NETs) are an umbrella term for various malignancies with histologic similarities, often identified with “dense core granules.” NETs are relatively rare, constituting ~0.5% of all diagnosed malignancies [1]. According to the SEER program, the age-adjusted annual incidence of NETs arising from jejunum and ileum is 0.67 per 100 000, and incidence and prevalence have been increasing over the past several decades due to increased awareness and improved recognition. However, due to their rarity and varied clinical presentations, NETs remain poorly understood [2]. NETs can have different primary sites, including the lungs, small intestines, and large intestines, leading to varying prognoses. Tumors that originate in the pancreas tend to have the worst prognosis. Although rare, NETs constitute a significant portion of primary small bowel neoplasms. Types can be further divided into well-differentiated, poorly differentiated, functioning, and nonfunctioning, with functioning tumors leading to the symptoms of the more well-known Carcinoid Syndrome [3]. The heterogeneity of the term “neuroendocrine tumors” has led to confusion, but the treatment of these tumors requires multidisciplinary care. We aim to educate the public regarding this rare disease to promote early detection and discuss treatment options to improve prognosis, as most NETs in the early stages are highly curable.

## Case presentation

A 72-year-old male was referred for surgical consultation of a ventral hernia. The patient had a past medical history of colon cancer status post colon resection 11 years ago with subsequent hernia repair 10 years ago. The patient noted a ventral hernia 6 months ago associated with left lower quadrant pain. He denied any nausea, vomiting, or changes in bowel movement. Significant family history includes colon cancer (maternal grandmother) and lung cancer (sister).

On examination, his vital signs were stable. A gastrointestinal exam was notable for the palpable weakness of the anterior abdominal wall with a definite diastasis component and a possible fascial defect.

**Figure 1:**
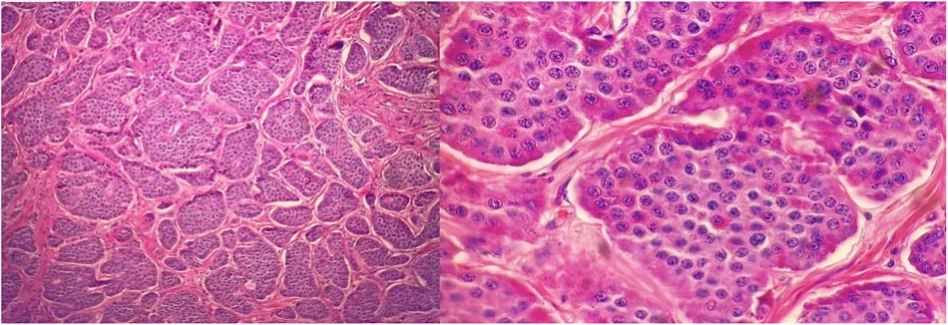
Well differentiated NETs involving mucosa, submucosa, muscularis propria of jejunum at 200× (left) and 600× (right) magnification.

At the time of the initial surgical consultation, the patient had already undergone a computed tomography (CT) scan of the abdomen and pelvis, demonstrating diastasis recti and slight herniation of mesenteric fat superior to the umbilicus. Six months later, he underwent elective diagnostic laparoscopic incisional hernia repair. Conversion to laparotomy was subsequently performed due to extensive adhesions with incorporated mesh to the small bowel wall. During postoperative day 1, leakage of clear to slimy green drainage was found to saturate his abdominal binder, likely due to delayed small bowel injury, which necessitated an emergency exploratory laparotomy. During this operation, an enterectomy was performed due to the incorporated mesh to the small bowel. During this exploration, numerous calcified lesions within the small bowel were noted to be scattered throughout the second half of the small bowel. The pathology report revealed numerous Grade 1 metastatic well-differentiated NET involving mucosa, submucosa, and muscularis propria (Fig. 1). The tumors also involve one out of seven lymph nodes. The tumors were categorized as stage III, pT2, and pN1. The immunohistochemistry report showed 0% of cells are positive for PD-L1 (22C3), and the tumor cells are negative for tropomyosin receptor kinase (TRK) protein expression.

His postoperative recovery was uneventful. After 4 weeks post laparotomy, he followed up with his oncologist. The positron emission tomography (PET) scan showed a general physiologic activity distribution in the thorax, abdomen, and pelvis. There is a small, modestly fluorodeoxyglucose (FDG) avid node in the left iliac chain with a standardized uptake value (SUV) max of 2.85 of undetermined origin. At this time, his oncologist recommended surveillance with CT imaging annually for 10 years.

## Discussion

Due to the heterogeneity of the term NETs, we will limit the terms of this discussion to those in the small bowel, as is this case. Diagnosing NETs is challenging. These tumors are often incidentally found when patients undergo evaluation for other disease processes, as they are usually asymptomatic. Even when patients are symptomatic, the clinical presentation is often nonspecific. Although some hereditary conditions, including multiple endocrine neoplasias, predispose individuals to develop this disease, most NETs occur sporadically.

Our patient was classified as having numerous well-differentiated Grade 1 tumors as per WHO classification for Neuroendocrine Neoplasms with 1 mitosis per 2 MM2. Grade 1 neoplasms have <2 mitotic counts. His staging was III. Treatment guidelines vary depending on the primary tumor’s location, grade, and stage. In general, if able, low-grade tumors are surgically resected, while unresectable tumors are treated with somatostatin analogs. High-grade tumors or patients presenting with metastatic disease are treated with platinum-based chemotherapy [1]. Upon receipt of the pathology report, our patient was informed of the non-resectable nature of the disease due to the extent of small bowel disease involving over ½ the small bowel. A follow-up PET scan after resection showed a physiologic distribution of activity in the thorax, abdomen, and pelvis. Since the tumors were slow growing, DOTATATE-PET/CT, which is more sensitive to NETS, is unlikely to show any visible disease given the negative initial PET scan. According to NCCN guidelines, unless there is a visible resectable tumor, an asymptomatic low-burden tumor should be managed by surveillance with abdominal and pelvic multiphasic CT every 3–12 months. In our case, the patient will undergo CT surveillance annually every 10 years per his oncologist’s recommendation following the NCCN guideline.

Overall, our patient received treatment similar to current guidelines. Current ENETS guidelines recommend imaging every 6–12 months with more frequent imaging for Grade 3 tumors [4].

NETs can present as carcinoid syndrome when functioning tumors secrete endogenous substances like serotonin. These patients will present with additional symptoms such as diarrhea, wheezing, and signs of heart disease. This presentation often occurs in patients already with metastatic disease. Although most jejunal and ileal tumors are nonfunctioning, up to 20% can present with carcinoid tumors. Common clinical manifestations will include abdominal pain, diarrhea, and obstructive symptoms. This location has a more unfavorable prognosis due to lower detection rates and advanced spread by the time of diagnosis [2]. This case further shows the importance of education and keeping NETs in differential diagnoses when treating elderly patients, especially those with small bowel obstruction and nonspecific symptoms.

## Conflict of interest statement

None declared.

## Funding

None declared.

## Data availability

The data underlying this article are available in the article and in its online supplementary material.

## Consent

Patient consent for study inclusion obtained.

## Guarantor

G.R. is the guarantor of this article.

## References

[1] Oronsky B, Ma PC, Morgensztern D, et al. Nothing but NET: a review of neuroendocrine tumors and carcinomas. Neoplasia 2017;19:991–1002. 29091800 10.1016/j.neo.2017.09.002 PMC5678742

[2] Xavier S, Rosa B, Cotter J. Small bowel neuroendocrine tumors: from pathophysiology to clinical approach. World J Gastrointest Pathophysiol 2016;7:117–24. 26909234 10.4291/wjgp.v7.i1.117 PMC4753177

[3] Raphael MJ, Chan DL, Law C, et al. Principles of diagnosis and management of neuroendocrine tumours. CMAJ 2017;189:E398–404. 28385820 10.1503/cmaj.160771 PMC5359105

[4] Larouche V, Akirov A, Alshehri S, et al. Management of small bowel neuroendocrine tumors. Cancers (Basel) 2019;11:1395. 31540509 10.3390/cancers11091395 PMC6770692

